# Evaluation of Traditional Indian Antidiabetic Medicinal Plants for Human Pancreatic Amylase Inhibitory Effect *In Vitro*

**DOI:** 10.1155/2011/515647

**Published:** 2010-09-23

**Authors:** Sudha Ponnusamy, Remya Ravindran, Smita Zinjarde, Shobha Bhargava, Ameeta Ravi Kumar

**Affiliations:** ^1^Institute of Bioinformatics and Biotechnology, University of Pune, Ganeshkhind, Pune 411007, India; ^2^School of Biotechnology, Amrita Vishwa Vidyapeetham, Kollam, Kerala 690525, India; ^3^Department of Zoology, Molecular Embryology Laboratory, University of Pune, Pune 411007, India

## Abstract

Pancreatic *α*-amylase inhibitors offer an effective strategy to lower the levels of post prandial hyperglycemia via control of starch breakdown. Eleven Ayurvedic Indian medicinal plants with known hypoglycemic properties were subjected to sequential solvent extraction and tested for *α*-amylase inhibition, in order to assess and evaluate their inhibitory potential on pancreatic *α*-amylase. Analysis of 91 extracts, showed that 10 exhibited strong Human Pancreatic Amylase (HPA) inhibitory potential. Of these, 6 extracts showed concentration dependent inhibition with IC_50_ values, namely, cold and hot water extracts from *Ficus bengalensis* bark (4.4 and 125 *μ*gmL^−1^), *Syzygium cumini* seeds (42.1 and 4.1 *μ*gmL^−1^), isopropanol extracts of *Cinnamomum verum* leaves (1.0 *μ*gmL^−1^) and *Curcuma longa* rhizome (0.16 *μ*gmL^−1^). The other 4 extracts exhibited concentration independent inhibition, namely, methanol extract of *Bixa orellana* leaves (49 *μ*gmL^−1^), isopropanol extract from *Murraya koenigii* leaves (127 *μ*gmL^−1^), acetone extracts from *C. longa* rhizome (7.4 *μ*gmL^−1^) and *Tribulus terrestris* seeds (511 *μ*gmL^−1^). Thus, the probable mechanism of action of the above fractions is due to their inhibitory action on HPA, thereby reducing the rate of starch hydrolysis leading to lowered glucose levels. Phytochemical analysis revealed the presence of alkaloids, proteins, tannins, cardiac glycosides, flavonoids, saponins and steroids as probable inhibitory compounds.

## 1. Introduction

Diabetes mellitus is a carbohydrate metabolism disorder of endocrine system due to an absolute or relative deficiency of insulin secretion, action, or both [1]. The disorder affects more than 100 million people worldwide and by 2030 it is predicted to reach 366 million. The most prevalent form both in the global and Indian scenario is the noninsulin dependent diabetes mellitus (NIDDM type 2) which is associated with elevated postprandial hyperglycemia [2]. 

Pancreatic *α*-amylase (E.C. 3.2.1.1), is a key enzyme in the digestive system and catalyses the initial step in hydrolysis of starch to maltose and finally to glucose. Degradation of this dietary starch proceeds rapidly and leads to elevated post prandial hyperglycemia (PPHG). It has been shown that activity of Human Pancreatic *α*-amylase (HPA) in the small intestine correlates to an increase in post-prandial glucose levels, the control of which is therefore an important aspect in treatment of diabetes [3]. Hence retardation of starch digestion by inhibition of enzymes such as *α*-amylase would play a key role in the control of diabetes. However, the discovery of specific high-affinity inhibitors of pancreatic *α*-amylase for the development of therapeutics has remained elusive. Inhibitors currently in clinical, use for example, acarbose, miglitol, and voglibose, are known to inhibit a wide range of glycosidases such as *α*-glucosidase and *α*-amylase. Because of their nonspecificity in targeting different glycosidases, these hypoglycemic agents have their limitations and are known to produce serious side effects. Therefore, the search for more safer, specific, and effective hypoglycemic agents has continued to be an important area of investigation with natural extracts from readily available traditional medicinal plants offering great potential for discovery of new antidiabetic drugs [4, 5]. 

While plant derivatives with purported hypoglycemic properties have been used in folk medicine and traditional healing systems, very few of these traditional anti-diabetic plants have received proper scientific or medical scrutiny despite recommendations by World Health Organization (WHO). Ayurveda and other Indian traditional approaches have described more than 800 plants in the Indian subcontinent, known to possess antidiabetic potential. These require to be effectively studied and in fact only few of them have been characterized for their mechanistic actions [6–9]. Plants such as *Barringtonia racemosa, Phyllanthus amarus, *and so forth, have been tested for the presence of pancreatic *α*-amylase inhibitors from various regions of the world [10–15]. Most of them have been tested on porcine pancreatic *α*-amylase (PPA) and salivary amylase while reports on their effect on human pancreatic amylase (HPA), if any, are scarce.

In this study, we have selected medicinal plants from the Indian Ayurvedic system with known hypoglycemic or antidiabetic activity, in an attempt to screen for new inhibitors for HPA. The selections of these plants were based on their traditional usage and also taking into consideration previous studies that have demonstrated their antidiabetic properties (Table 1), thus providing a preliminary screening assessment [16–26]. However, no known reports of these plants as HPA inhibitors exist. Sequential solvent extracts of *Azadirachta indica A. Juss., Bixa orellana L., Bougainvillea spectabilis Willd*., *Cinnamomum verum J. S. Presl, Curcuma longa L*., *Ficus bengalensis L., Ficus racemosa L.*, *Momordica charantia L., Murraya koenigii L. Spreng., Syzygium cumini L. Skeels*, and *Tribulus terrestris L.* were tested for the presence of PPA and HPA inhibitors. The lead extracts have also been subjected to preliminary kinetics on HPA to ascertain the type of inhibition as well as for phytochemical analysis to determine the probable inhibitory compounds present. 

## 2. Methods

### 2.1. Chemicals

Starch, porcine pancreatic *α*-amylase (PPA), methanol, isopropanol, acetone, methyl-butyl-tertiary ether, cyclohexane, and dimethylsulfoxide (DMSO) were purchased from SRL Pvt. Ltd, Mumbai, India. 3,5-dinitrosalicylicacid (DNSA) was obtained from HiMedia Laboratories, Mumbai, India. Human pancreatic *α*-amylase (HPA) and acarbose were purchased from Sigma Aldrich, USA. All other chemicals procured were from local manufacturer and were of AR grade.

### 2.2. Plant Material

In this study, anti-diabetic plants from the Indian sub-continent were selected on the basis of known ethno botanical/traditional Ayurvedic literature. Plants were obtained from Pune city and nearby areas of the Western Ghats, India, in the months of August–January. The plants, their voucher numbers (V. No.), the part(s) used, and the hypoglycemic properties as seen from literature search are listed in Table 1. A specimen of each plant was submitted to Botanical Survey of India (BSI), Pune, India, for authentication. The plants were separated into their various parts, namely., roots, stem, leaves, seeds and so forth, washed with tap water, air-dried in shade, and processed immediately as mentioned below.

### 2.3. Preparation of Plant Extracts

The air-dried plant material (60–100 g) was crushed with liquid nitrogen, powdered, and successively extracted in polar to nonpolar solvent on an increasing degree of non-polarity [27]. The different extracts obtained sequentially were with cold water, hot water, methanol, isopropanol, acetone, methyl-butyl-tertiary ether, and cyclohexane. This kind of sequential extraction was performed taking into consideration the fact that traditional methods of preparing herbal formulations are mainly aqueous. Also, aqueous extracts contain peptides, proteins, or glycans, which would otherwise be denatured by organic solvents and high-temperature extraction. Distilled water was added to the crushed material in a ratio of 1 : 4 (w/v) and kept at 30°C (24 h) and 55°C (2 h) at 130 rpm for cold-and hot-water extracts, respectively. For each solvent, the extract was filtered, centrifuged, and the residue collected for subsequent solvent extraction. The organic solvents were added in a ratio of 1 : 3 (w/v) and refluxed with the residue for 3 h at their respective boiling temperatures. Each extract was filtered and stored at −20°C.

### 2.4. *α*-Amylase Inhibition and Kinetic Studies

PPA was used for preliminary screening of *α*-amylase inhibitors from the extracts. The inhibition assay was performed using the chromogenic DNSA method [28]. The total assay mixture composed of 500 *μ*L of 0.02 M sodium phosphate buffer (pH 6.9 containing 6 mM sodium chloride), 0.04 units of PPA solution, and extracts at concentration from 0.1–1.5 mg mL^−1^(w/v) were incubated at 37°C for 10 min. After pre-incubation, 500 *μ*L of 1% (v/v) starch solution in the above buffer was added to each tube and incubated at 37°C for 15 min. The reaction was terminated with 1.0 mL DNSA reagent, placed in boiling water bath for 5 min, cooled to room temperature, diluted, and the absorbance measured at 540 nm. The control reaction representing 100% enzyme activity did not contain any plant extract. To eliminate the absorbance produced by plant extract, appropriate extract controls were also included. One unit of enzyme activity is defined as the amount of enzyme required to release one micromole of maltose from starch per min under the assay conditions. 

For the determination of the inhibitor concentration at which 50% inhibition of enzyme activity occurs (IC_50_), the assay was performed as above except that the inhibitor/plant extract concentrations were varied from 0.1–150 *μ*g. Acarbose was used as a positive control at a concentration range of 6.5 *μ*g–32.8 *μ*g. The IC_50_ values were determined from plots of percent inhibition versus log inhibitor concentration and calculated by logarithmic regression analysis from the mean inhibitory values. The IC_50_ values were defined as the concentration of the extract, containing the *α*-amylase inhibitor that inhibited 50% of the PPA or HPA activity. Other quantifiers were defined as follows:

%Relative enzyme activity = (enzyme activity of test/enzyme activity of control) ∗ 100;

%inhibition in the *α*-amylase activity = (100 − % relative enzyme activity).

 For kinetic experiments involving concentration independent inhibition, the inhibitor/extracts were taken at their IC_50_ values and incubated with HPA while the substrate (starch) concentration was varied from 0.5–5 mg mL^−1^ and reaction allowed to proceed as mentioned above. Kinetic analysis was performed based on Lineweaver-Burk double reciprocal plots and the kinetic parameters calculated using Origin 6.0 software from Originlab, USA. 

### 2.5. Preliminary Phytochemical Analysis

Qualitative phytochemical analyses of the extracts were performed according to Parekh and Chanda [29]. Alkaloids were detected by Mayer's reagent, while for cardiac glycosides the Keller-Kiliani test was carried out. Steroids were detected using the Liebermann-Burchard test. Bradford reagent was used to detect the proteins while the frothing test was used for detection of saponins.

### 2.6. GCMS Analyses

Analyses of the organic extracts exhibiting ≥50% inhibition on the PPA or HPA activity were performed on a GCMS-MS (Varian 4000) gas chromatograph equipped with DB 5 ms capillary column (30 m × 0.25 mm ID). Helium was the carrier gas with flow rate of 1 mL/min, the injector mode—split (1 : 60), the injection volume 1 *μ*L, the temperature program used is as follows: 50°C (3 min), then increased to 280°C at 15°C /min, held at 280°C (10 min) and temperature scan, *m*/*z *35–800. Appropriate solvent controls were also run. The identification of the components was based on the comparison of their mass spectra with those of NIST-Wiley 2008 library.

### 2.7. Statistical Analysis

All experiments were performed in 3 different sets, with each set in triplicates. The data are expressed as mean ± SEM. Statistical analysis was performed for analysis of variance (ANOVA) followed by *F*-test using SPSS 11.5. Those *P*-values ≤  .05 were considered as significant. 

## 3. Results

### 3.1. Screening of Plant Extracts on *α*-Amylase Inhibition

**Figure 1:**
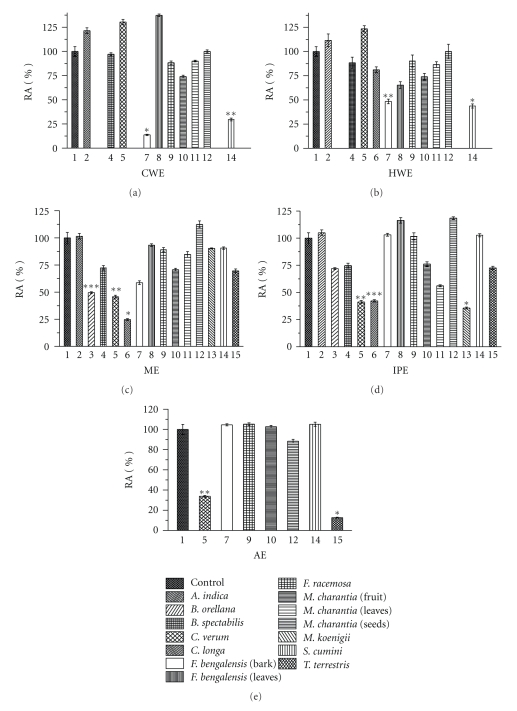
The percent relative enzyme activity (RA %) of porcine pancreatic *α*-amylase (PPA) on inhibition with different extracts. (a) Cold-water extracts (CWE). (b) Hot-water extracts (HWE). (c) Methanol extracts (ME). (d) Isopropanol extracts (IP). (e) Acetone extracts (AE) of the listed plants. Pure porcine pancreatic *α*-amylase serves as control. The data is indicated as the mean ± SEM; (*n* = 3). The students *F*-test was used and the bars with different asterisks (***, **, *) show significant difference with respect to control (*P* <  .05).

The plants from the Indian sub-continent exhibiting potential hypoglycemic properties were sequentially extracted with polar to non-polar solvents and a total of 91 extracts obtained. It should be noted that while generally cold-and hot-water extracts are most commonly used in the traditional method of preparing medicines in Ayurveda, chances of missing out on bioactive principles with better amylase inhibitory potential from less-polar solvents are high. Hence the rationale for performing extractions from polar to non-polar solvents is not only to confirm and validate the inhibitory activity, if found, in any of the aqueous extractions performed in the traditional manner but also to search for newer and higher specific affinity inhibitors in less-polar solvents. PPA was used as a target enzyme for screening of inhibitory activity from the above mentioned ninety-one plant extracts. The control reaction representing 100% enzyme activity was 0.21 U/mL for PPA and 0.25 U/mL for HPA. Extract samples dissolved in DMSO contained as a final yield of 1.5 mg mL^−1^ of the dried extract and the enzyme activity of both PPA and HPA was not affected by DMSO at the concentration used. Appropriately diluted plant extract was used for enzyme inhibition assay and the activity obtained with each extract was normalized to percent relative activity (Figure 1) from which the percent inhibition was calculated. Less than 50% PPA inhibition was observed for extracts of *A. indica *(leaves),* B. spectabilis *(leaves),* F. bengalensis *(leaves), *F. racemosa *(fruit), *M*. *charantia *(leaves, fruit, and seeds) as well as for extracts of MTBE and cyclohexane. Some of the extracts were found to marginally activate PPA. Of the rest, 12 extracts exhibited significant inhibition (≥50%) on PPA enzyme activity. The most significant inhibitory activity was obtained with the aqueous extracts of *F. bengalensis *bark (0.38 mg mL^−1^ and 0.14 mg mL^−1^ for cold and hot water, resp.), *S. cumini *(0.38 mg  mL^−1^ and 0.13 mg mL^−1^ for cold and hot water), methanol extracts of *B. orellana *(0.04 mg mL^−1^), *C.longa *(1.5 mg mL^−1^), *C. verum *(2.23 mg mL^−1^), isopropanol extracts of *C. longa* (0.01 mg mL^−1^), *C. verum* (0.06 mg mL^−1^), *M. koenigi *(0.05 mg mL^−1^) and acetone extracts of *C. longa* (0.02 mg mL^−1^) and* T. terrestris *(0.33 mg mL^−1^). On checking the stability of these extracts over a period of time, all the extracts except for *C. verum *methanol extract were stable even after a period of two months. Thus, further experiments were carried out with 11 stable extracts (excluding *C. verum *methanol extract) exhibiting PPA inhibition ≥50%. These extracts exhibited significant inhibition (*P*  ≤  .05) with IC_50_ values ranging between 10 and 920 *μ*gmL^−1^. *C. longa* and *M. koenigii* isopropanol extracts and *S. cumini *cold water extract showed the best IC_50_ value of 10 *μ*gmL^−1^ against PPA. The known PPA inhibitor, acarbose, taken as a positive control exhibited an IC_50_ value of 10.2 *μ*gmL^−1^. Those extracts exhibiting ≥50% inhibition for PPA were taken as lead extracts and were further tested for inhibition studies against HPA. 

### 3.2. Extracts Exhibiting ≥50% Inhibition on Human Pancreatic Amylase (HPA) Activity

**Figure 2:**
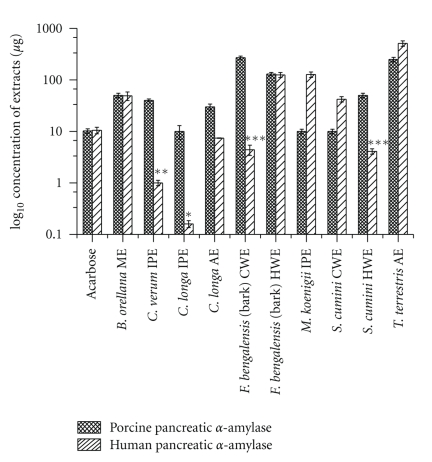
IC_50_ values of the extracts exhibiting ≥50% inhibition on Porcine pancreatic *α*-amylase and Human pancreatic *α*-amylase enzyme activity. The data is calculated as the mean ± SEM; (*n* = 3). The students F-test was used and the bars with different asterisks (***, **, *) show significant difference with respect to control (P  <  .05). Acarbose is taken as the standard *α*-amylase inhibitor.

Of the 11 lead extracts obtained, *C. longa* methanol extract was not tested against HPA since the concentration required for inhibiting PPA (920 *μ*gmL^−1^) was high. For the other 10 extracts, differing IC_50_ values were obtained for HPA as compared to PPA, with values ranging from 0.16 *μ*gmL^−1^ to 511 *μ*gmL^−1^ (Figure 2). Of these, *B. orellana* methanol extract and *F. bengalensis* hot-water extract showed similar IC_50_ values for HPA as seen for PPA, while *M. koenigii* isopropanol, *S. cumini *cold-water, and *T. terrestris *acetone extracts required higher concentrations for 50% HPA inhibition as compared to PPA. However, five extracts, namely, isopropanol extracts of *C. longa* l (0.16 *μ*gmL^−1^) and *C. verum* (1.0 *μ*gmL^−1^), cold-water extract of* F. bengalensis* (4.4 *μ*gmL^−1^), and hot-water extracts of *S. cumini * (4.1 *μ*gmL^−1^) could inhibit HPA at much lower concentrations than PPA. Acarbose inhibited HPA with an IC_50_ value of 10.5 *μ*gmL^−1^. Thus, these crude extracts could inhibit HPA at much lower concentrations than even acarbose and would therefore be good candidates to test for high-affinity inhibitors. For this, inhibitor kinetic studies were performed to determine the type of inhibition.

### 3.3. Kinetic Studies

**Figure 3:**
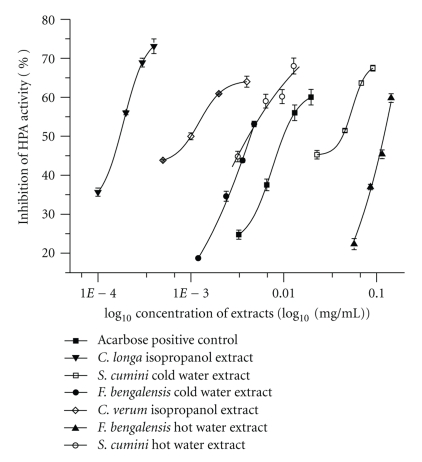
Human Pancreatic *α*-amylase inhibition (%) of different extracts at varying concentrations. The data is indicated as the mean ± SEM; (*n* = 3). (*P* <  .05).

The inhibitory assays were performed using extracts at different dilutions in order to check for concentration dependent or independent inhibition. Of the 10 plant extracts studied, 6 extracts, namely, *C. longa* (isopropanol),* C. verum* (isopropanol), *F. bengalensis* (cold and hot water), and *S. cumini *(cold and hot water) exhibited concentration dependent (competitive) inhibition. Plots of percent inhibition versus log concentration (Figure 3) of extracts showed typical sigmoidal dose response curves with 50% inhibition at concentrations 0.16 *μ*gmL^−1^, 1.0 *μ*gmL^−1^, 4.4 *μ*gmL^−1^, 125 *μ*gmL^−1^, 42.1 *μ*gmL^−1^, and 4.1 *μ*gmL^−1^, respectively, for HPA. The lowest IC_50_ value for HPA was noted for *C. longa* isopropanol extract at 0.16 *μ*gmL^−1^. Many of these extracts were found to competitively inhibit HPA at much lower concentrations than the commercially used inhibitor acarbose (10.5 *μ*gmL^−1^).

**Figure 4:**
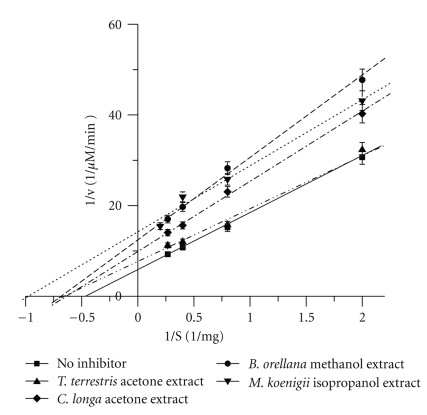
Lineweaver-Burk Plot of extracts exhibiting concentration independent inhibition on Human pancreatic *α*-amylase enzyme activity. The data is indicated as the mean ± SEM; (*n* = 3); (*P* <  .05).

On the other hand, 4 extracts, namely, *B. orellana* (methanol), *C. longa *(acetone), *M. koenigii, *(isopropanol), and *T. terrestris *L. (acetone) extracts exhibited concentration independent inhibition for HPA. A preliminary study for the probable mode of inhibition was performed using Michaelis-Menten kinetics at their IC_50 _value with varying substrate concentrations. The double reciprocal Lineweaver-Burk plot showed a decrease in both *V*_max_ and *K*_m_ values for all the 4 extracts studied, thereby suggesting an uncompetitive type of inhibition (Figure 4). For control HPA, that is, without inhibitor, using starch as the substrate, the apparent *V*_max_ and *K*_m_ values of 0.17 *μ*Mmin^−1^, 2.12 mg, respectively, were obtained. These kinetic parameters decreased to 0.08 *μ*Mmin^−1^ and 1.36 mg for *B. orellana*, 0.10 *μ*Mmin^−1^ and 1.61 mg for *C. longa*, 0.07 *μ*Mmin^−1^ and 1.02 mg for* M. koenigii,* and 0.14 *μ*Mmin^−1^ and 1.71 mg for* T. terrestris, *respectively. 

### 3.4. Phytochemical and GC-MS Analysis

Preliminary qualitative phytochemical analysis was performed to determine the probable type of compounds present in the extracts causing HPA inhibition. The results showed the presence of different types of active constituents such as proteins, cardiac glycosides, saponins, alkaloids, flavonoids, and so forth. (Table 2).

Using the Wiley-NIST library, GC-MS identification of the compounds based on the retention time, peak area, molecular mass, and molecular formula are shown in Table 3. 

## 4. Discussion

**Figure 5:**
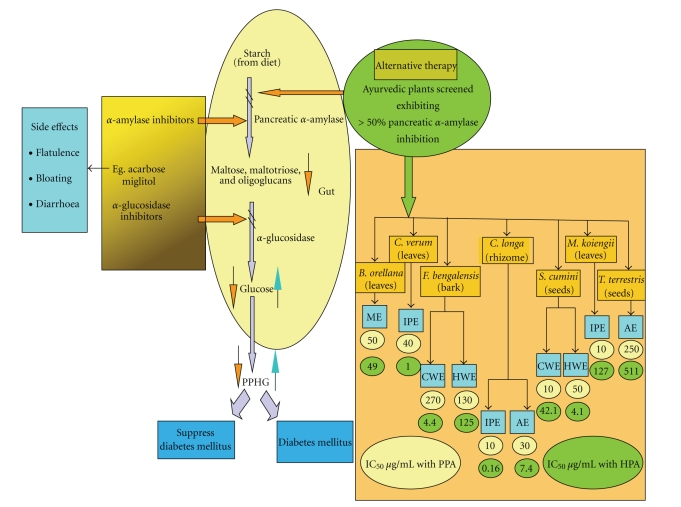
Pancreatic *α*-amylase inhibition by Indian medicinal plant extracts as a potential antidiabetes mechanism. Double bar marks (*∖* *∖*) indicate inhibition of amylase activity leading to a reduction of maltose, oligosaccharide and glucose concentration. ME: Methanol extract; AE: Acetone extract; IPE: Isopropanol extract; CWE: Cold-water extract; HWE: Hot-water extract.

The aim of the present study was to investigate the HPA inhibitory activity from medicinal plants known in the Indian Ayurvedic system for their anti-diabetic properties. To date no reports of compounds responsible for HPA inhibition from these plants exist in the literature and we report here for the first time their inhibitory on HPA. Many of the plants chosen in this study are used by the Indian population not only for food purposes but also form a part of the local pharmacopoeia for the treatment of diabetes. Our results indicate that retardation of starch hydrolysis by inhibition of HPA activity by some of these extracts leads to a reduction in glucose concentrations (Figure 5). Of the 11 plants and their 91 extracts tested, 10 exhibited significant inhibition of HPA, suggesting that they contain compounds capable of HPA inhibition. Of these, 4 are aqueous extracts while 6 are extracts of less-polar solvents. Preliminary phytochemical analysis to indicate the kind of compounds present in these extracts suggests the occurrence of proteins/peptides and polyphenols in cold-and hot-water extracts while tannins, alkaloids, flavonoids, and saponins are found in non-polar extracts. Flavonoids and polyphenolics have been reported to contribute to hypoglycemic activity [30]. 

*B. orellana* methanol extract exhibited concentration independent inhibition with an IC_50_ value of 49 *μ*gmL^−1^. The major components identified in this extract were found to be *β*-tocopherols and vitamin E which are known to be present in *B. ornella* leaves [31]. 

*C. verum* inhibited HPA in a concentration-dependent manner with a low IC_50_ value of 1.0 *μ*gmL^−1^. The major components identified were naphthalene, 1, 2, 3, 4-tetrahydro-1, 1, 6-trimethyl, eugenol, and 4-acetoxycinnamic acid and have been previously reported [32, 33].

*C. longa* is known to contain curcuminoids, glycosides, terpenoids, and flavonoids [34]. Maximal inhibition of the enzyme (HPA) was obtained with *C. longa *isopropanol extract at a low concentration of 0.16 *μ*gmL^−1^ in a competitive manner and with acetone extract at 7.4 *μ*gmL^−1^ in a concentration independent manner. The probable compounds in *C. longa *isopropanol extract were podocarpic acid, curlone, and cinnamic acid while those in acetone extract were curlone, 3-cyano-7-hydroxy-4-methylcoumarin, and 5-amino-2-hydroxybenzoic acid. 

*F. bengalensis *bark extract showed HPA inhibitory activity in the aqueous fraction with an IC_50_ value of 4.4 *μ*gmL^−1^ in a concentration-dependent manner. The major phytochemical components in the aqueous extract were found to be alkaloids, proteins, tannins, flavonoids, and saponins while cardiac glycosides and steroids were found to be absent. A dimethoxy derivative of leucocyanidin 3-O-beta-D-galactosyl cellobioside isolated from the bark of *F. bengalensis* Linn. has been shown to decrease blood sugar levels significantly both in normal and moderately diabetic rats [35].

The compounds identified in *M. koenigii* isopropanol extract were cyclohexanone 2-methyl-5-(1-methylethenyl), 2, 3, 5, 6 tetrachlorohydroquinone, and Vitamin E which have been reported in leaves by other investigators [36, 37]. The extract was able to inhibit HPA in a concentration independent manner with an IC_50_ value of 127 *μ*gmL^−1^. Kinetic analysis also indicated that this extract resulted in the best decrease in both *K*_m_ and *V*_max_ values to 1.02 mg and 0.07 *μ*Mmin^−1^ as compared to control, namely, 2.12 mg and 0.17 *μ*Mmin^−1^.

*S. cumini* is known to contain proteins, tannins, an alkaloid jambosine, a glycoside antimellin, and fatty acids [38]. The extracts exhibiting concentration-dependent HPA inhibitory activity were cold-and hot-water extracts with an IC_50_ value of 42.1 and 4.1 *μ*gmL^−1^, respectively. It is most likely that the bioactive component could probably be a protein, a glycoside, or a polyphenol.

Furostanol and spirostanol saponins, flavonoids, alkaloids, steroids, carbohydrates, and amides have been isolated from *T. terrestris *[39]. In our study, the acetone extract-inhibited HPA activity in a concentration independent manner with a high IC_50_ value of 511 *μ*gmL^−1^. The compounds identified in this extract were ethyl crotonate and sorbinose.

These findings suggest that inhibition of HPA activity, leading to retardation of starch hydrolysis is one of the mechanisms through which the plants tested in this study could be exhibiting their hypoglycemic effect. Modulation of HPA activity by compounds in these extracts would thus eventually lead to a lowering of post-prandial blood glucose levels (Figure 5). Presence of HPA inhibitor blocks the normal pathway of conversion of dietary starch to maltose, maltotriose, and oligosaccharides and then to glucose in the gut which gets absorbed in the blood. Thus, glucose formation can be blocked in hyperglycemic conditions with pancreatic *α*-amylase inhibitors. This HPA inhibition could occur in a concentration dependent or independent manner depending upon the bioactive compound. The compounds/bioactive principle from these plants responsible for the HPA inhibition need to be isolated and characterized. The efficacy of HPA inhibition can be increased with the combination of these isolated components as shown by Said et al., where in herbal combination of four plants is more effective as antidiabetic traditional medicine [40]. 

## 5. Conclusions

This study highlights the HPA inhibitory activity of 10 plant extracts exhibiting promising leads as HPA inhibitory molecules and lends scientific support to their use in traditional Ayurvedic medicine. *B. orellana, F. bengalensis, *S*. cumini*, *C. verum, M. koenigii, C. longa*, and *T. terrestris* plants, which are known for their hypoglycemic property, were found to exhibit strong inhibitory action on HPA, even better than acarbose. The phytochemicals in the extracts causing this inhibition were identified as alkaloids, proteins, tannins, cardiac glycosides, flavonoids, saponins, and steroids. Studies to isolate the bioactive principle(s) from these extracts are required. In the recent years, the search for new molecules as potential *α*-amylase inhibitors with a high specific affinity has intensified. Thus, modulation of HPA activity through the therapeutic use of high-affinity plant derived inhibitors would be of considerable medical relevance in the treatment of diabetes. 

## Figures and Tables

**Table 1:** Plant sources and their traditional uses.

Plants name^a^	V. no^b^	Family	Parts used	Hypoglycemic and medicinal properties	Ref.
*Azadirachta indica A. Juss.* (Neem)	GMAI3	Meliaceae	Leaves	Antihyperglycemic activity, increase in glucose uptake and glycogen deposition, inhibits activity of epinephrine on glucose metabolism resulting in utilization of peripheral glucose. Does not alter cortisol concentration.	[17, 18]
*Bixa orellana L.* (Sindhuri)	BOSP1	Bixaceae	Leaves	Hypoglycemic activity by lowering blood glucose by stimulating peripheral utilization of glucose.	[19]
*Bougainvillea spectabilis Willd.* (Bougainvilla)	MARCKBS2	Nyctaginaceae	Leaves	Hypoglycemic effect is by D-pinitol which exerts insulin-like effect and inhibits *α*-glucosidase.	[20]
*Cinnamomum verum J.S.Presl* (Cinnamon)	CIZYS1	Lauraceae	Leaves	Hypoglycemic activity is by enhancing insulin activity, increasing lipid metabolism and antioxidant status, capillary function.	[21]
*Curcuma longa L.* (Turmeric)	SUCL1	Zingiberaceae	Rhizome	Hypoglycemic, hypolipidemic, and antioxidant property. Decreased influx of glucose in polyol pathway, increasing NADPH/NADP ratio and increased activity of glucose peroxidase.	[22]
*Ficus bengalensis L.* (Banyan)	GMFB1	Moraceae	Leaves, Bark	Stimulates insulin secretion from beta cells inhibits insulin degradative process.	[23]
*Ficus recemosa L.* (Umbar)	GMFR2	Moraceae	Fruit pulp	Hypoglycemic activity by *β*-sitosterol isolated was found to lower the blood glucose level.	[24]
*Momordica charantia L.* (Karela,) (Bitter gourd)	MARCK1	Cucurbitaceae	Leaves, fruit, seeds	Hypoglycemic effect by inhibition of glucose-6-phosphatase and fructose-1-6-biphosphatase in liver and stimulation of hepatic glucose-6-phosphate dehydrogenase.	[25]
*Murraya koenigii L.* Spreng (Curry Leaves)	SP-2	Rutaceae	Leaves	Increases glucogenesis and decreases glycogenolysis and gluconeogenesis.	[26]
*Syzygium cumini L. Skeels* (Jamun)	SPSC2	Myrtaceae	Seeds	Reduces blood glucose level, increase in serum insulin level, exhibits insulinase activity. Hypoglycemic activity mediated through insulin release mechanism, glycogen content and hepatic glucokinase, hexokinase, glucose-6-phosphate, and phosphofructokinase levels in diabetic mice.	[4, 17]
*Tribulus terrestris L.* (Gokshura)	SUTT1	Zygophyllaceae	Seeds	Hypoglycemic activity by inhibiting oxidative stress.	[16]

^a^The common Indian name for the plant is given in brackets.

^b^Voucher numbers as given by Botanical Survey of India, Pune.

**Table 2:** Qualitative phytochemical analysis of the extracts exhibiting ≥50% inhibition on PPA and HPA enzyme activity.

Plant species	Alkaloids	Proteins	Tannins	Cardiac glycosides	Flavonoids	Saponins	Steroids
*B. orellana L. *	−	+	+	+	−	+	+
(ME)							
*C. verum *	+	+	+	+	−	+	+
*J. S. Presl* (IPE)							
*C. longa L. *	+	−	+	−	+	+	+
(IPE)							
*C. longa L. *	+	−	+	−	+	−	+
(AE)							
*F. bengalensis L. *	+	+	+	−	+	+	−
(CWE)							
*F. bengalensis L. *	+	+	−	−	+	+	−
(HWE)							
*M. koenigii L.*	+	−	+	+	−	+	+
*Spreng* (IPE)							
*S. cumini L. *	+	+	+	−	+	+	−
*Skeels.* (CWE)							
*S. cumini L. *	+	+	+	−	+	+	−
*Skeels.* (HWE)							
*T. terrestris L. *	+	−	−	−	+	+	−
(AE)							

+: Detected; −: Not detected; CWE: Cold-water extract; HWE: Hot-water extract; ME: Methanol extract; IPE: Isopropanol extract; AE: Acetone extract.

**Table 3:** GCMS identification of compounds in organic solvent extracts of plants inhibiting HPA enzyme activity.

Plant	Name of the compound	Molecular formula	Molecular weight	Area %	Retention time (min)
*B. orellana L. *	*β*-tocopherol	C_28_H_48_O_2_	416	38.85	24.29
(ME)	Vitamin E	C_29_H_50_O_2_	430	50.17	25.96
*C. verum *	Naphthalene,1,2,3,4-tetrahydro-1,1,6-trimethyl	C_13_H_18_	174	32.20	6.29
*J. S. Presl* (IPE)	Eugenol	C_10_H_12_O_2_	164	2.57	11.13
	4-acetoxycinnamic acid	C_11_H_10_O_4_	206	47.02	12.55
					
*C. longa L. *	Podocarpic acid	C_17_H_22_O_3_	274	48.21	13.69
(IPE)	Curlone	C_15_H_22_O	218	19.53	13.95
	Cinnamic-acid	C_9_H_8_O_2_	148	7.03	14.79
*C. longa L. *	3-Cyano-7-hydroxy-4-methylcoumarin	C_11_H_7_NO_3_	201	42.95	13.66
(AE)	Curlone	C_15_H_22_O	218	17.19	13.97
	5-amino-2-hydroxybenzoic acid	C_7_H_7_NO_3_	153	9.574	17.82
					
*M. koenigii L.*	Cyclohexanone, 2-methyl-5-(1-methylethenyl)-	C_10_H_16_O	152	18.54	14.8
*Spreng* (IPE)	2,3,5,6-tetrachlorohydroquinone	C_6_H_2_Cl_4_O_2_	247	51.85	24.21
	Vitamin E	C_29_H_50_O_2_	430	10.56	25.94
					
*T. terrestris L. *	Sorbinose	C_6_H_12_0_6_	180	10.27	14.27
(AE)	Ethyl crotonate	C_6_H_10_O_2_	114	71.36	16.96

ME: Methanol extract; IPE: Isopropanol extract; AE: Acetone extract.
